# Incident stroke in patients with Alzheimer’s disease: systematic review and meta-analysis

**DOI:** 10.1038/s41598-021-95821-x

**Published:** 2021-08-12

**Authors:** João Pinho, Miguel Quintas-Neves, Imis Dogan, Kathrin Reetz, Arno Reich, Ana Sofia Costa

**Affiliations:** 1 Department of Neurology, University Hospital RWTH Aachen, Pauwelsst. 30, 52074 Aachen, Germany; 2 Neuroradiology Department, Hospital de Braga, Braga, Portugal; 3 JARA Institute Molecular Neuroscience and Neuroimaging, Juelich Research Center GmbH and RWTH Aachen University, Aachen, Germany; 4 Life and Health Sciences Research Institute (ICVS), School of Medicine, University of Minho, Braga, Portugal; 5 ICVS/3B´s-PT Government Associate Laboratory, Braga/Guimarães, Portugal

**Keywords:** Cerebrovascular disorders, Stroke, Alzheimer's disease

## Abstract

Vascular mechanisms are increasingly recognized in the pathophysiology of Alzheimer’s disease (AD), but less is known about the occurrence of stroke in AD patients. We aimed to quantify the risk of stroke in patients with AD and compare the incidence rates (IR) of stroke in individuals without AD. Systematic search of Embase and MEDLINE between 1970 and 2020. Inclusion criteria: reports with ≥ 50 patients with non-familial AD, which reported the occurrence of stroke (all types) and/or ischemic stroke and/or intracerebral hemorrhage (ICH) during follow-up. Meta-analyses of pooled data using random-effects model were performed. IR were calculated for each study. Incidence rate ratios (IRR) were calculated for studies presenting a control-group without AD. Among 5109 retrieved studies, 29 (0.6%) fulfilled the inclusion criteria, reporting a total of 61,824 AD patients. In AD patients the IR were 15.4/1000 person-years for stroke (all types), 13.0/1000 person-years for ischemic stroke and 3.4/1000 person-years for ICH. When compared to controls without AD, incidence rate for ICH in AD patients was significantly higher (IRR = 1.67, 95%CI 1.43–1.96), but similar for ischemic stroke. Incident stroke is not a rare event in AD population. AD is associated with an increased risk of intracerebral hemorrhage which warrants further clarification.

## Introduction

Stroke continues to be one of the leading causes of disability-adjusted life years and of global mortality^1^. Its primary prevention represents a major epidemiological challenge^2,3^ and has implications for other diseases, namely dementia^4^. Alzheimer’s disease (AD) is the most prevalent neurodegenerative disease leading to dementia. Its respective associated burden of disease has increased in the last decades in high income countries mainly due to aging of the population^5^. Increasing age is one of the major risk factors for both stroke and AD, therefore, it is no surprise that both conditions frequently co-occur^6^. However, the pathophysiological relationship between the neurodegenerative processes in AD and vascular dysfunction is complex^4,7^. Even though the focus of many studies has been incident cognitive decline and dementia after the occurrence of stroke^8^, there is increasing evidence suggesting that microangiopathic mechanisms may have a role in the pathogenesis of AD^4^. Age-related mechanisms which contribute to the direct involvement of the brain parenchyma in the AD neurodegenerative process may also play a role in the early vascular dysregulation observed in late-onset AD^9^. In addition to that, the presence of both amyloid microangiopathy and non-amyloid cerebral microangiopathy in pathological studies was demonstrated to be highly frequent in AD patients and more frequent in AD than in other neurodegenerative diseases^10^, and supports the pivotal role that the neurovascular unit may play in the pathophysiology of AD^11^. The importance and significance of amyloid vascular deposition in AD patients is a current matter of debate and entails relevant implications concerning not only possible pathophysiological mechanisms, such as dysfunction of perivascular amyloid clearance pathways, but also contribution to further brain damage through microangiopathic and macroangiopathic complications, such as white matter changes, cortical microinfarcts and stroke^12,13^. The fact that midlife classical vascular risk factors, which are the cause for arteriolosclerotic cerebral microangiopathy, are also associated with increased amyloid brain deposition is a further sign of the complex relationship between vascular and neurodegenerative processes^14^. A question that remains open is whether patients with AD, due to the aforementioned vascular involvement, present a higher lifetime risk of experiencing cerebral macrovascular complications such as ischemic stroke and intracerebral hemorrhage (ICH). One previous meta-analysis studied the incidence of ischemic stroke and ICH in patients with AD, but presented several methodological limitations, such as the inclusion of studies with partially overlapping populations, inclusion of a study with patients with other causes of dementia and the exclusion of studies which did not report stroke subtype^15^. In the present work we aimed to overcome these limitations and to reduce sources of bias by improving the study inclusion criteria. Our goal was to study patients with AD and calculate the risk of incident stroke of all types, ischemic stroke and ICH, and to compare the risk of stroke in this population of patients with the risk of stroke in a matched population without AD. We performed a systematic review of available literature and conducted a meta-analysis of studies which reported incident stroke in AD patients.

## Methods

We conducted a systematic review and meta-analysis according to the Preferred Reporting Items for Systematic Reviews and Meta-Analyses (PRISMA) guidelines^16^. The PRISMA checklist is presented in the Supplementary Material. This study was not registered and there is no published study protocol.

### Search strategy

We used Embase to search in Embase and MEDLINE records, using a comprehensive search term to identify human studies published in English, German, French, Portuguese, Spanish or Italian between 1970 and October 8, 2020, which reported patients with AD and occurrence of ischemic stroke, ICH and/or stroke. The search strategy and search term are further detailed in the Supplementary Material. We additionally reviewed the references of relevant articles to search for additional studies.

### Eligibility criteria

We included studies which: reported ≥ 50 patients with a diagnosis of AD; reported a retrospective or prospective follow-up period; specifically reported the occurrence of ischemic stroke and/or non-traumatic ICH and/or stroke (all types) during the follow-up period. We excluded: studies which did not report criteria for AD diagnosis; studies reporting only patients with other causes of dementia or with unspecified cause of dementia; studies reporting incident stroke in patients with genetic/familial forms of AD or in patients with Down syndrome; studies in which incident stroke numbers were not retrievable specifically for patients with AD. Both clinical and research criteria for AD diagnosis were accepted for study inclusion. Randomized or single-arm therapeutic trials which fulfilled the above criteria were included. For therapeutic trials of anti-β-amyloid immunotherapies, which are associated with the development of amyloid related hemorrhages, only the placebo groups were included in the meta-analysis. Among studies with overlapping populations, the publication reporting a larger study population was selected, except for studies which reported different outcomes (for example, one article reporting ischemic stroke whereas another article reporting ICH), in which case both articles were included. Title and abstract screening were performed by three authors (JP, MQN and ASC). Studies that met the predefined Patient (adults), Exposure (diagnosis of AD), Comparison (if available, control group without AD) and Outcome (occurrence of incident stroke) (PECO) criteria were selected. Selection of the final studies for inclusion in the meta-analysis was performed independently by two authors (JP and ASC), and disagreements were settled by consensus after joint review of the full text.

### Data extraction

Two authors (JP and ASC) extracted key information of the studies according to a pre-planned form, and recorded it in two separate databases, which were later compared and corrected for inconsistencies. The following variables were collected: type of study, methods for diagnosis of AD, population size, age, sex, AD severity, Mini Mental State Examination score, vascular risk factors, antithrombotic therapy, follow-up, incident ischemic stroke, incident ICH, localization of incident ICH, incident stroke (all types). The occurrence of stroke (all types) included ischemic stroke, ICH and unspecified type of stroke. If studies reported both ischemic stroke and ICH, stroke (all types) was defined as the sum of both events. If follow-up in person-years was not reported in the article, total person-years was estimated based on mean follow-up or planned follow-up. For randomized trials which reported follow-up time for patients who dropped out, this information was used to calculate total person-years. Incident stroke in age- and sex-matched control-groups without AD was additionally collected from studies which presented a control population.

To assess the quality of the all included studies we used the Newcastle–Ottawa Quality Assessment Scale for Cohort Studies^17^. The quality of randomized controlled studies was assessed as if the studies were observational cohort studies for our research question. The quality of evidence was classified using the GRADE tool^18^.

### Statistical analysis

Using total follow-up in person-years and incident ischemic stroke, incident ICH and incident stroke (all types) for each study, we performed random effects meta-analyses of single incidence rates (IR) by using the metarate function contained in the meta package in R version 4.0.2 (R Project for Statistical Computing). For studies which also reported incident ischemic stroke, incident ICH and/or incident stroke (all types) for a matched control-group without AD, we conducted a random effects meta-analysis of incidence rate ratios (IRR) by using the metainc function. A continuity correction of 0.1 in studies with no incident stroke was applied. The inverse variance method was used for study weighing. We quantified study heterogeneity by calculating Cochrane’s *Q*-test, I^2^ and *τ*^2^. Risk of publication bias in the meta-analyses for incidence rate ratios was analysed qualitatively using funnel plots, the low number of included studies precluded the application of an asymmetry test. All incidence rates and incidence rate ratios were derived directly from the number of events of interest occurring during follow-up and from the total number of person-years of follow-up reported in each study. We analysed the effect of antithrombotic therapy in the incidence of ischemic stroke and of ICH in patients with AD by calculating the IRR in studies reporting these events separately for groups treated and not treated with antiplatelets and/or anticoagulants. In order to address the impact of possible bias associated with inclusion of randomized controlled studies primarily not design to detect the occurrence of stroke, we performed the same random effects meta-analyses of observational cohort studies only.

## Results

**Figure 1:**
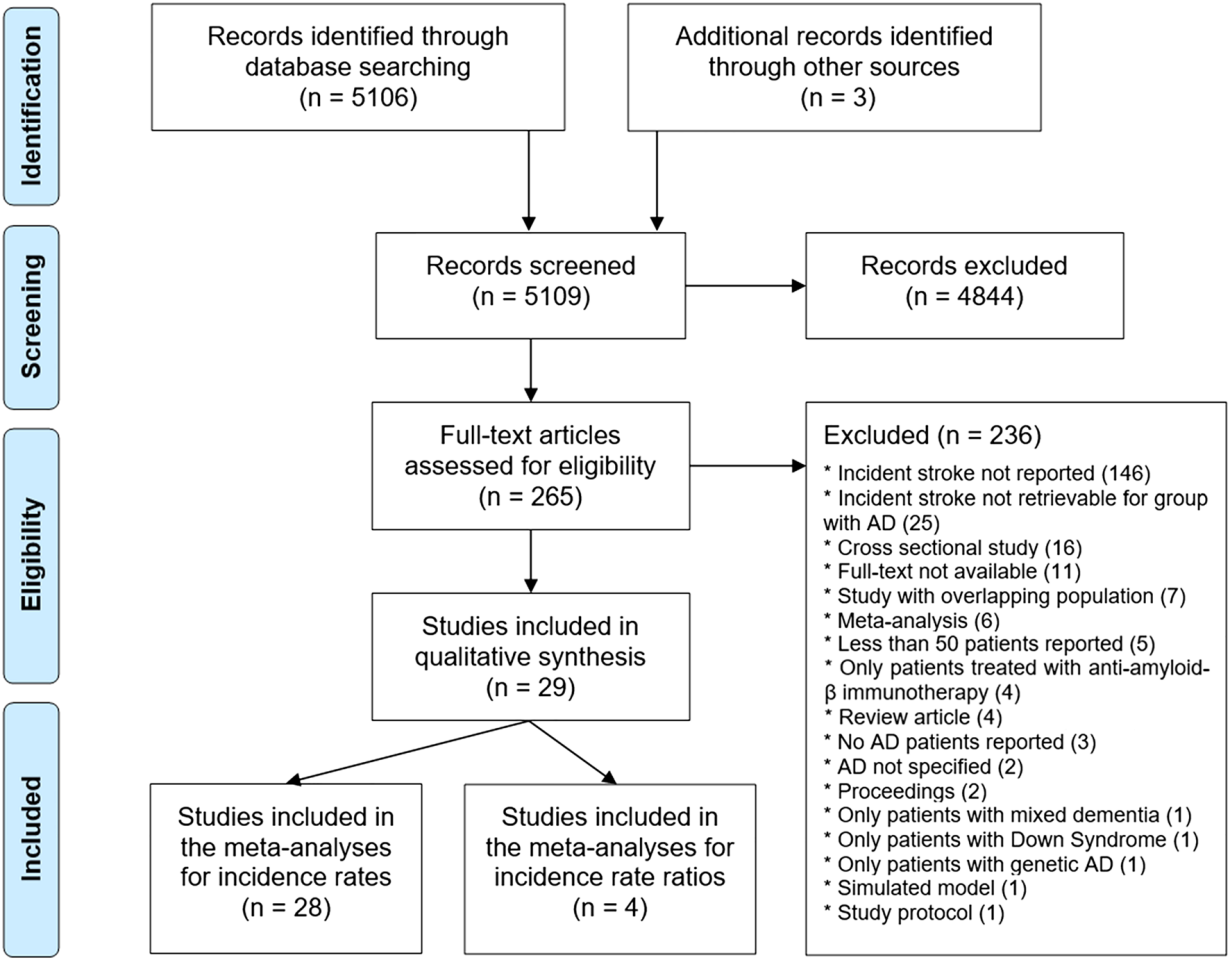
Preferred Reporting Items for Systematic Reviews and Meta-Analyses (PRISMA) diagram.

The electronic search retrieved 5106 articles and 3 additional articles were identified through review of the references of relevant articles (Fig. 1). After title and abstract screening, 4844 articles were excluded (of which 2590 were review papers and 535 were animal studies), and the full-texts of 265 papers were reviewed. Of these, 29 articles reported incident ischemic stroke and/or incident ICH and/or incident stroke (all types) in patients with AD and were included in the meta-analyses. Table 1 summarizes the main characteristics of the included studies, and describes the PECO criteria for each study. One of the articles^19^ reported incident ICH in a population of AD patients which partially overlapped with another older study which additionally reported incident ischemic stroke^20^. In this case, only incident ICH from the first study, and incident ischemic stroke and incident stroke (all types) from the second study were collected to avoid double counting in the meta-analysis. Another pair of articles reported overlapping populations^21,22^. The article reporting the larger population was included for the meta-analyses of incident rates^21^ and the article also reporting age- and sex-matched controls without AD was included for the meta-analyses of incident rate ratios^22^. Twelve articles reported incident ischemic stroke, 18 articles reported incident ICH and 21 articles reported incident stroke (all types). Overall, 28 articles were included in the meta-analyses of IR, with a total of 61,824 patients with AD, 2102 incident ischemic strokes, 623 incident ICH and 2810 incident strokes (all types). Four articles which reported incident stroke for a matched control population without AD were included in the meta-analyses of IRR (35,261 AD patients, 46,289 matched controls). A mean age of $\ge$ 75 years at AD diagnosis for the studied population was reported in 13/26 studies. In 7 of the 8 studies which reported disease severity, patients had mild to moderate dementia. The fact that the majority of studies did not report disease severity and almost no study included patients in severe stages of AD precluded a further analysis of stroke incidence according to disease severity. A mean Mini Mental State Examination score lower than 20 was reported in 17/22 studies. In the majority of studies (22/29) no information concerning antithrombotic therapy was available. Information concerning ICH localization was not retrievable from any article.

**Table 1:** Detailed characteristics of the studies included in the meta-analysis.

Study	Design	AD diagnosis	Number of patients	Mean age (years)	Female (%)	AD severity	Mean MMSE	Hypertension (%)	Anti-thrombotic therapy (%) *	Follow-up	Control group without AD	Incident ischemic stroke (n)	Incident intracerebral hemorrhage (n)	Incident stroke, all types (n) ^†^
Lee et al. 2020^19‡^	Retrospective cohort	DSM-IV	1648	76.2	57.6	NA	NA	62.4	50.0	6767 person-years	16,480	NA	39	NA
Taipale et al. 2017^21^^§^	Retrospective cohort	NINCDS-ADRDA; DSM-IV	45,050	NA	63.7	NA	NA	31.2	17.0	122,136 person-years	–	1832	510	2397
Vandenberghe et al. 2016^37^^¶^	RT	NINCDS-ADRDA	759	70.0	59.2	Mild to moderate	20.2	NA	NA	1.5 years (per protocol)	–	NA	2	NA
Benedictus et al. 2015^38^	Prospective cohort	NINCDS-ADRDA; NIAAA	301	71.2	42.0	NA	21.0	22.8	40.5	1500 patient-years	–	12	5	23
Cummings et al. 2015^39^	RT	NIAAA	220	77.8	57.3	NA	16.6	NA	NA	10 weeks (per protocol)	–	NA	NA	1
Salloway et al. 2014^23^^¶^	RT	NINCDS-ADRDA	972	72.1	53.3	Mild to moderate	20.8	NA	NA	1.5 years (per protocol)	–	8	1	10
Chi et al. 2013^20^^‡^	Retrospective cohort	NINCDS-ADRDA; DSM-IV	980	NA	59.9	NA	NA	72.8	NA	4 years (mean)	4900	139	21	160
Tolppanen et al. 2013^22^^§^	Retrospective cohort	NINCDS-ADRDA; DSM-IV	27,170	NA	NA	NA	NA	NA	NA	91,510 person-years	23,638	1217	276	1541
Imfeld et al. 2013^40^	Retrospective cohort	Algorithm based on diagnostic codes from primary care database	6443	80.6	69.5	NA	NA	35.9	NA	15,688 person-years	6171	74	43	117
Trzepacz et al. 2013^41^	RT	NINCDS-ADRDA; DSM-IV-TR	132	77.5	50.8	NA	16.9	NA	NA	3 months (per protocol)	–	NA	1	NA
Epstein et al. 2012^42^	Prospective cohort	NINCD-ADRDA	184	75.4	47.8	NA	NA	73.0	NA	2.6 years (mean)	–	NA	NA	2
Lee et al. 2011^43^	RT	NINCDS-ADRDA; DSM-IV	887	73.6	66.6	NA	16.4	NA	NA	24 weeks (per protocol)	–	NA	NA	2
Li et al. 2010^44^	Prospective cohort	NINCDS-ADRDA; DSM-IV	324	72.9	70.4	NA	15.5	35.5	NA	5 years	–	32	9	41
Farlow et al. 2010^45^	RT	NINCDS-ADRDA; DSM-IV-TR	261	77.2	57.9	Mild to moderate	18.3	NA	NA	3 months (per protocol)	–	NA	NA	1
Grossberg et al. 2009^46^	RT	NINCDS-ADRDA; DSM-IV	870	73.5	66.0	NA	16.5	NA	NA	28 weeks (per protocol)	–	NA	NA	4
Richard et al. 2009^47^	RT	NINCD-ADRDA	123	76.4	56.9	NA	21.9	NA	36.6	2 years (per protocol)	–	NA	3	NA
Clerici et al. 2009^48^	Prospective cohort	NINCD-ADRDA	451	77.0	71.6	Moderate to severe	9.0	NA	NA	6 months (per protocol)	–	NA	NA	1
Kessler et al. 2008^49^	RT	NINCD-ADRDA	68	69.5	47.1	Mild	NA	NA	NA	1 year (per protocol)	–	1	1	2
de Jong et al. 2008^50^	RT	NINCD-ADRDA	51	72.4	64.7	Mild to moderate	19.6	NA	NA	1 year (per protocol)	–	1	NA	NA
AD2000 CG, 2008^51^	RT	DSM-IV	310	75 (median)	62.9	NA	19 (median)	19.7	50.3	3 years (per protocol)	–	NA	4	20
Bakchine et al. 2008^52^	RT	NINCDS-ADRDA; DSM-IV	470	73.8	63.2	Mild to moderate	18.9	NA	NA	6 months (per protocol)	–	NA	2	NA
Mintzer et al. 2007^53^	RT	DSM-IV	480	82.5	80.0	NA	12.4	NA	NA	10 weeks (per protocol)	–	2	1	6
Howard et al. 2007^54^	RT	NINCD-ADRDA	259	84.6	84.6	NA	8.1	NA	NA	12 weeks (per protocol)	–	NA	NA	1
Soininen et al. 2007^55^	RT	NINCDS-ADRDA; DSM-IV	425	73.6	54.8	Mild to moderate	19.6	28.7	34.6	1 year (per protocol)	–	NA	1	10
Regan et al. 2006^56^	Prospective cohort	NINCDS-ADRDA; DSM-IV	167	81.7	72.5	NA	14.1	NA	NA	1.5 years	–	NA	NA	7
Silvestrini et al. 2006^57^	Prospective cohort	NINCDS-ADRDA	53	70.3	47.0	Mild to moderate	17.5	49.0	11.0	1 year	–	0	0	0
Schneider et al. 2006^58^	RT	NINCDS-ADRDA; DSM-IV	421	77.9	55.8	NA	15.0	NA	NA	36 weeks (per protocol)	–	NA	NA	5
Seltzer et al. 2004^59^	RT	NINCDS-ADRDA; DSM-IV-TR	153	74.0	53.6	Mild	24.2	NA	NA	24 weeks (per protocol)	–	NA	1	NA
Imbimbo et al. 2000^60^	RT	NINCDS-ADRDA	342	72.6	64.9	NA	17.7	NA	NA	6 months (per protocol)	–	1	NA	NA

*AD* Alzheimer’s disease, *RT* randomized trial, *NA* not available, *NINCD-ADRDA* National Institute of Neurological and Communicative Diseases and Stroke/Alzheimer's Disease and Related Disorders Association, *DSM-IV* Diagnostic and Statistical Manual of Mental Disorders, fourth edition, *DSM-IV-TR* Diagnostic and Statistical Manual of Mental Disorders, fourth edition, text revision, *MMSE* Mini Mental State Examination.

*Antithrombotic therapy includes antiplatelet therapy and/or anticoagulation.

^†^Incident stroke (all types) includes ischemic stroke, intracerebral hemorrhage and unspecified stroke type.

^‡,§^Studies with overlapping population.

^¶^Only patients in the non-interventional group were included.

The primary aim of most of the included studies was not to report incident stroke in AD patients (e.g., therapeutic randomized trials with cognitive progression as primary outcome). This is the reason why only 4 studies achieved a maximal score in the Newcastle–Ottawa Quality Assessment Scale for Cohort Studies (Supplementary Table S1). The design of most of the studies was randomized trial (n = 18), followed by prospective cohort (n = 6) and retrospective cohort (n = 5). This study provides Class II evidence for the incidence of stroke in the AD population and the overall quality of evidence according to GRADE is low to moderate.

### Incidence rates of stroke in AD patients

**Figure 2:**
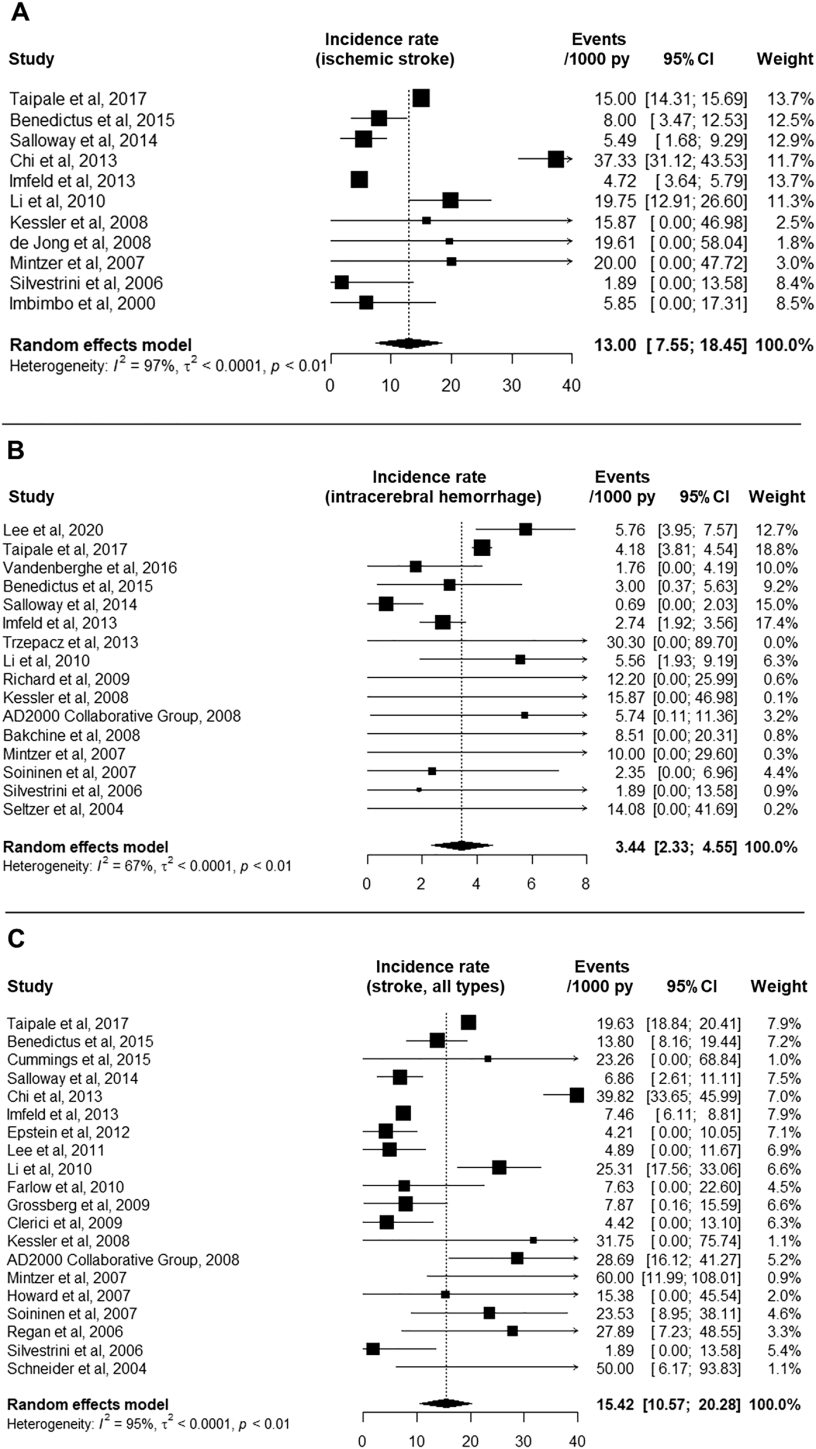
Forest plot of the random effects meta-analysis for incident ischemic stroke (**A**), incident intracerebral hemorrhage (**B**) and incident stroke, all types (**C**) in patients with Alzheimer’s disease.

Figure 2 summarizes the pooled IR of ischemic stroke, ICH and stroke (all types) among patients with AD. Pooled IR for ischemic stroke was 13.0 per 1000 person-years (95% confidence interval [95%CI] 7.6–18.5), for ICH was 3.4 per 1000 person-years (95%CI 2.3–4.6) and for stroke (all types) was 15.4 per 1000 person years (95%CI 10.6–20.3). Significant study heterogeneity was found in the meta-analyses of IR of ischemic stroke and stroke (all types), as depicted by I^2^ values ≥ 95% (p < 0.01). The meta-analysis of studies reporting incident ICH presented a moderate study heterogeneity (I^2^ = 67%, p < 0.01) and the lowest *Q* (*Q* = 44.9 Vs 337.4 for stroke of all types and 372.2 for ischemic stroke). The exclusion of the randomized controlled trials did not change the results significantly (Supplementary Fig. S1). Meta-analyses of the 5 studies which reported incident ischemic stroke and/or incident ICH separately for AD patients treated and not treated with antiplatelets/anticoagulation revealed no significant effect of the antithrombotic therapy in the incidence of ischemic stroke or ICH, but the IRRs had relatively large confidence intervals (Supplementary Fig. S2).

### Incidence rate ratios of stroke in AD patients (compared to a matched-control group without AD)

Only four studies reported incidence rate for stroke both in AD patients and in matched controls without AD^19,20,22,23^. Although two of these studies had overlapping populations^18,23^, only the most recent one^19^ was used to calculate IRR for ICH.

**Figure 3:**
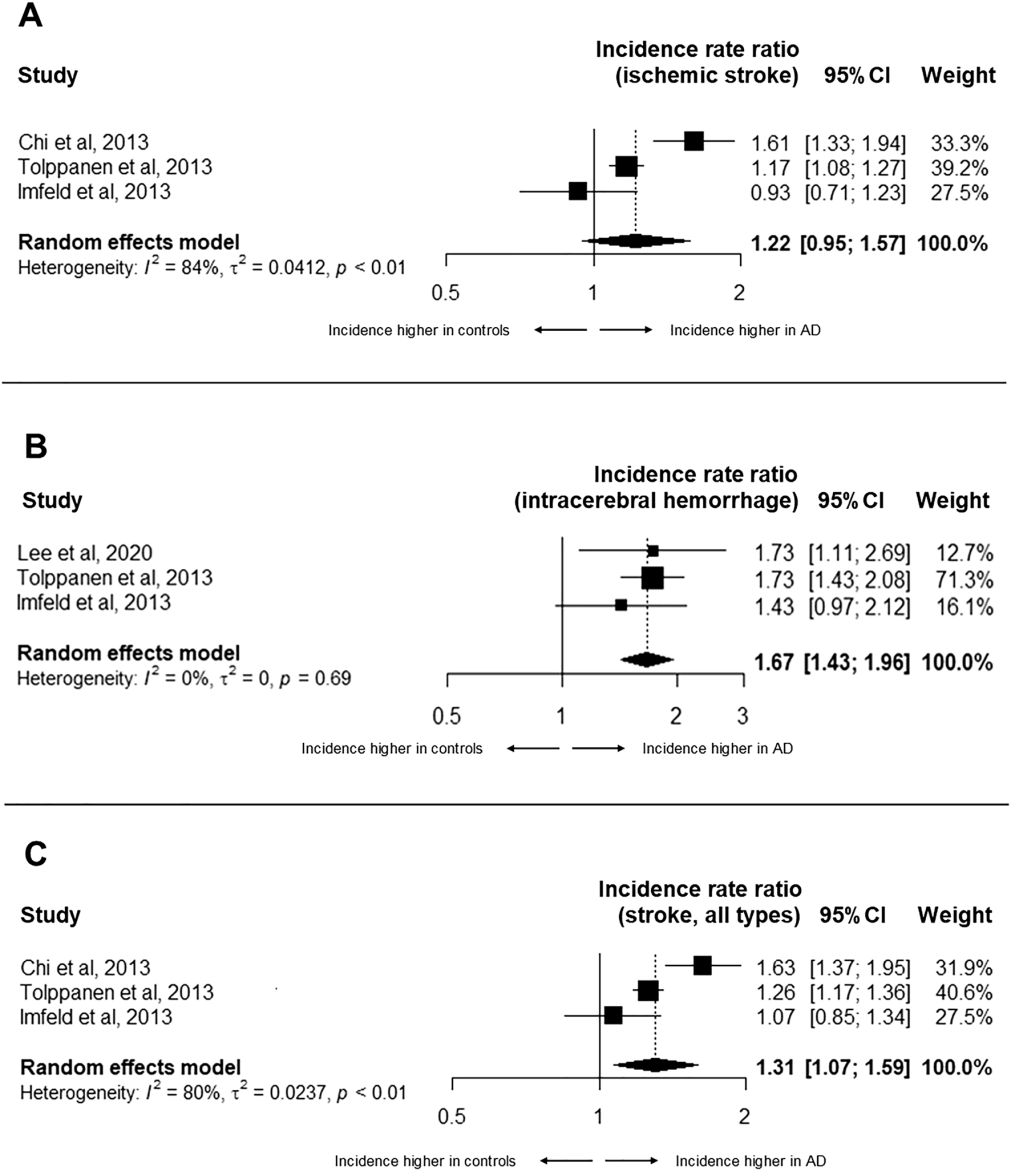
Forest plot of the random effects meta-analysis for incidence rate ratios for ischemic stroke (**A**), intracerebral hemorrhage (**B**) and incident stroke, all types (**C**) in patients with Alzheimer’s disease compared with matched controls without Alzheimer’s disease.

In each of the four studies, groups of AD patients and control-groups without AD were matched for age, sex and for additional variables which varied in each study (e.g., vascular risk factors, comorbidities, malignancy). Figure 3 summarizes the incidence rate ratios for ischemic stroke, ICH and stroke (all types). Incidence rate for ischemic stroke in AD patients was not significantly different from matched controls without AD (IRR = 1.22, 95% CI 0.95–1.57, p = 0.123). Incidence rates for both ICH and stroke (all types) was significantly higher in patients with AD when compared to matched controls, as depicted by IRR of 1.67 (95%CI 1.43–1.96, p < 0.001) and 1.31 (95%CI 1.07–1.59, p = 0.008), respectively. Significant study heterogeneity was found in the meta-analyses of incidence rate ratios for ischemic stroke and stroke (all types), but not for ICH (I^2^ = 0%, p = 0.69). The lowest *Q* value was found for the meta-analysis of IRR for ICH (*Q* = 0.74 Vs 9.9 for stroke of all types and 12.9 for ischemic stroke). Funnel plots for the three studies suggested no significant publication bias (Supplementary Figs. S3–S5).

## Discussion

We systematically reviewed the evidence available in the literature reporting incident stroke in patients with AD. The main findings consist of the pooled incidence rates of stroke in patients with AD: 15.4 strokes of all types per 1000 person-years (95%CI 10.6–20.3), 13.0 ischemic strokes per 1000 person-years (95%CI 7.6–18.5) and 3.4 ICH per 1000 person-years (95%CI 2.3–4.6). We found a significant risk of bias and heterogeneity among the studies included in the meta-analyses, which were lower for the pooled incidence rates of ICH. Therefore, these results must be interpreted with caution. It is believed that in low and middle-income countries the vascular contribution to dementia will become a major public health problem due to the increasing incidence of vascular risk factors^4^. Simultaneously, even though the incidence of stroke in high-income countries has been steadily decreasing in the last decades, the aging of the population results in an increasing absolute number of first-ever strokes^24^. This evidence is particularly worrying for the population with dementia, in which the occurrence of stroke is expected to be accompanied by a significant increase in disability and health care needs. The knowledge of stroke incidence in patients with AD helps to plan health care resources allocation and to design specific prevention programs for this group of patients. A previous meta-analysis by Waziry and collaborators of studies reporting stroke in AD patients showed similar incidence rates for ischemic stroke (14.0/1000 person-years) and ICH (3.4/1000 person-years) within confidence intervals found in our study^15^. However, this previous meta-analysis presents several limitations, namely the inclusion patients from two pairs of studies with partially overlapping populations, the inclusion of one study which reported patients with any cause of dementia and the exclusion of studies which did not report stroke subtype. We aimed to overcome these limitations by avoiding double counting of patients from studies with overlapping populations, by including only studies reporting the criteria used for AD diagnosis, by including studies reporting stroke of all types and by including studies which did not report only first-ever strokes.

We found that the incidence rate of stroke of all types in patients with AD was higher than in matched controls without AD. This difference was mainly driven by a higher incidence rate of ICH in AD patients (IRR = 1.67, 95%CI 1.43–1.96), without a significant difference in ischemic stroke incidence between AD patients and matched controls. Even though only four studies with a multivariable matched population without AD were included in the meta-analysis, risk of bias and study heterogeneity were low for the calculation of IRR for ICH. Similar results were found in the aforementioned meta-analysis by Waziry and collaborators and in a meta-analysis by Zhu and collaborators^25^, which reported a slight underestimation of IRR for both ICH and ischemic stroke, probably due to the different methodology employed.

Several mechanisms may underlie the possible association between stroke and AD. The fact that the incidence of both diseases clearly increase with age reflects that both neurodegeneration and vascular disease are decisively influenced by ageing processes such as genomic instability, mitochondrial dysfunction, cellular senescence, altered intercellular communication and oxidative stress^26,27^. There is also evidence which suggests that neurodegenerative processes in AD and vascular processes may influence each other. Cerebrovascular effects of β-amyloid include direct vasoconstriction of cerebral vessels, reactive oxygen species-mediated persistent pericyte contraction with consequent impairment of cerebral autoregulation and reduced cerebral blood flow. Pathological evidence of amyloid deposition in cerebral small vessels has been reported to occur in > 80% of patients with AD, with increasing severity of amyloid angiopathy in occipital lobes correlating with severity of AD pathology^28^. The partial overlap between AD and cerebral amyloid angiopathy^29^ could be the reason for the increased risk of ICH in AD patients. Unfortunately, no information concerning the location or etiology of ICH was available in the studies included in our meta-analysis. At the same time, there are some suggestions that ischemia may also promote AD pathology. Chronic cerebral hypoperfusion in animal models was shown to increase the size and amount β-amyloid plaques, possibly through a shift in the formation of aggregation-prone β-amyloid species^30^. Cerebral atherosclerosis, arteriolosclerosis and gross infarcts appear to be independently associated with clinically defined AD dementia^31^, and midlife vascular risk factors have been associated with increased amyloid deposition in florbetapir positron emission tomography^14^. However, there is an ongoing debate concerning this question, because other studies did not find an association between AD pathological changes and higher vascular risk profile^32^, midlife carotid atherosclerosis^33^ or intracranial atherosclerosis and stenosis^34^.

Our results concerning the lack of relevant effect of antithrombotic therapy on the incidence of ischemic stroke and of ICH in AD patients must be interpreted with caution given the small number of studies used to calculate the IRRs, heterogeneity of the study populations and relatively large associated confidence intervals. Further studies assessing the ischemic and bleeding risk in patients with AD treated with oral anticoagulants and with antiplatelets are warranted. Even though the risk of ICH appears to be higher in patients with AD when compared with patients with no AD, the absolute risk of ischemic stroke is higher than the risk of ICH. In this respect, it should be noted that our study is unsuitable to provide recommendations concerning antithrombotic treatment in patients with AD.

The majority of included studies, given their original aims, lack a comprehensive clinical characterization of the AD population. Nonetheless, as derived from available data from AD clinical severity and MMSE score, most of the patients included in this meta-analysis are classified as mild to moderate dementia. Even though there is increasing evidence of vascular dysfunction in early stages of AD^35^, cerebral vascular lesion load increases with age because most of the vascular risk factors and other important causes for stroke, such as atrial fibrillation, are strongly correlated with age^10^. In addition to association with age, a correlation between the extension of microangiopathic changes and pathologically-defined cerebral β-amyloid/neurofibrillary tangle stages of AD, which were associated with the clinical severity of dementia, was demonstrated by Thal and collaborators^36^. Taking this evidence in consideration, it is probable that the incidence of ischemic stroke and ICH in advanced stages of AD may be even higher than the incidence we found. However, one must not rule out a possible effect of selective survival of AD patients with less vascular pathology until later stages of the disease, because of early cardiovascular death of AD patients with high risk vascular profile.

A strength of our study is that we used an inclusive search term, whereby a broad range of articles were considered, which on the other hand led to a high exclusion rate as most studies did not fulfil criteria for qualitative or quantitative data synthesis. Other strengths include the independent selection of studies performed by more than one author, exclusion of studies with overlapping populations, inclusion of studies reporting stroke of all types, relatively high number of total patients with AD included in the meta-analyses and systematic evaluation of the quality of the included studies. However, our study also presents limitations. Significant study heterogeneity is probably related to variability in identification of stroke and high variability of the follow-up periods, as well as in other important sociodemographic variables. There is a relative paucity of studies specifically designed to assess incident stroke in patients with AD, which is the reason why the overall quality of included studies was not optimal. Clinical randomized therapeutic trials consist of study settings which are designed to detect outcomes of interest and adverse events, but the relatively short follow-up periods in some trials, patient over-selection related to strict inclusion and exclusion criteria may impair generalization of the results concerning incident stroke in AD. Several questions remain to be answered, namely the risk of stroke depending on disease stage and severity, impact of deep and lobar microbleeds in the risk of both ischemic stroke and ICH in AD patients, characterization of location and etiology of ICH in AD patients, and short- and long-term outcome of stroke in AD patients.

In conclusion our study describes incidence rates of stroke of all types, ischemic stroke and ICH in patients with AD, and suggests that the risk of ICH may be higher in AD patients when compared to matched controls without AD. These findings are important to plan health care resources for patients with AD, they allow clinicians to consider stroke occurrence when predicting prognosis in patients with AD, provide insight for understanding the relationship of neurodegenerative processes and vascular changes in AD and call for better quality evidence regarding incidence of stroke in AD.

## Supplementary Information

Supplementary Information.
