# Supplementary material for: Incident stroke in patients with Alzheimer’s disease: systematic review and meta-analysis
Source: Sci Rep. 2021 Aug 12;11:16385. doi: 10.1038/s41598-021-95821-x (PMC8361108; doi:10.1038/s41598-021-95821-x)

Incident stroke in patients with Alzheimer's disease: systematic review and meta-analysis.

João Pinho, Miguel Quintas-Neves, Imis Dogan, Kathrin Reetz, Arno Reich, Ana Sofia Costa

SUPPLEMENTARY MATERIAL INDEX

PRISMA checklist ………………………………………. page 2-3

Search strategy ..……………………………………….. page 4

Supplementary Table 1 ………………………………… page 5

Supplementary Figure 1 ……………………………….. page 6

Supplementary Figure 2 ……………………………….. page 7

Supplementary Figure 3 ……………………………….. page 8

Supplementary Figure 4 ……………………………….. page 9

Supplementary Figure 5 ……………………………….. page 10

PRISMA checklist

Section/topic	#	Checklist item	Reported on page #
**TITLE**			
Title	1	Identify the report as a systematic review, meta-analysis, or both.	1
**ABSTRACT**			
Structured summary	2	Provide a structured summary including, as applicable: background; objectives; data sources; study eligibility criteria, participants, and interventions; study appraisal and synthesis methods; results; limitations; conclusions and implications of key findings; systematic review registration number.	2
**INTRODUCTION**			
Rationale	3	Describe the rationale for the review in the context of what is already known.	3
Objectives	4	Provide an explicit statement of questions being addressed with reference to participants, interventions, comparisons, outcomes, and study design (PICOS).	3
**METHODS**			
Protocol and registration	5	Indicate if a review protocol exists, if and where it can be accessed (e.g., Web address), and, if available, provide registration information including registration number.	3
Eligibility criteria	6	Specify study characteristics (e.g., PICOS, length of follow-up) and report characteristics (e.g., years considered, language, publication status) used as criteria for eligibility, giving rationale.	3-4
Information sources	7	Describe all information sources (e.g., databases with dates of coverage, contact with study authors to identify additional studies) in the search and date last searched.	3-4
Search	8	Present full electronic search strategy for at least one database, including any limits used, such that it could be repeated.	4, supp. material
Study selection	9	State the process for selecting studies (i.e., screening, eligibility, included in systematic review, and, if applicable, included in the meta-analysis).	4
Data collection process	10	Describe method of data extraction from reports (e.g., piloted forms, independently, in duplicate) and any processes for obtaining and confirming data from investigators.	4-5
Data items	11	List and define all variables for which data were sought (e.g., PICOS, funding sources) and any assumptions and simplifications made.	4-5
Risk of bias in individual studies	12	Describe methods used for assessing risk of bias of individual studies (including specification of whether this was done at the study or outcome level), and how this information is to be used in any data synthesis.	4, supp. material
Summary measures	13	State the principal summary measures (e.g., risk ratio, difference in means).	5
Synthesis of results	14	Describe the methods of handling data and combining results of studies, if done, including measures of consistency (e.g., I^2) for each meta-analysis.	5-6
Risk of bias across studies	15	Specify any assessment of risk of bias that may affect the cumulative evidence (e.g., publication bias, selective reporting within studies).	4
Additional analyses	16	Describe methods of additional analyses (e.g., sensitivity or subgroup analyses, meta-regression), if done, indicating which were pre-specified.	5-6

Section/topic	#	Checklist item	Reported on page #
**RESULTS**			
Study selection	17	Give numbers of studies screened, assessed for eligibility, and included in the review, with reasons for exclusions at each stage, ideally with a flow diagram.	6
Study characteristics	18	For each study, present characteristics for which data were extracted (e.g., study size, PICOS, follow-up period) and provide the citations.	6
Risk of bias within studies	19	Present data on risk of bias of each study and, if available, any outcome level assessment (see item 12).	6-7
Results of individual studies	20	For all outcomes considered (benefits or harms), present, for each study: (a) simple summary data for each intervention group (b) effect estimates and confidence intervals, ideally with a forest plot.	Figure 2
Synthesis of results	21	Present results of each meta-analysis done, including confidence intervals and measures of consistency.	6-7
Risk of bias across studies	22	Present results of any assessment of risk of bias across studies (see Item 15).	6-7
Additional analysis	23	Give results of additional analyses, if done (e.g., sensitivity or subgroup analyses, meta-regression [see Item 16]).	7
**DISCUSSION**			
Summary of evidence	24	Summarize the main findings including the strength of evidence for each main outcome; consider their relevance to key groups (e.g., healthcare providers, users, and policy makers).	8
Limitations	25	Discuss limitations at study and outcome level (e.g., risk of bias), and at review-level (e.g., incomplete retrieval of identified research, reporting bias).	8-10
Conclusions	26	Provide a general interpretation of the results in the context of other evidence, and implications for future research.	9-10
**FUNDING**			
Funding	27	Describe sources of funding for the systematic review and other support (e.g., supply of data); role of funders for the systematic review.	20

From: Moher D, Liberati A, Tetzlaff J, Altman DG, The PRISMA Group (2009). Preferred Reporting Items for Systematic Reviews and Meta-Analyses: The PRISMA Statement. PLoS Med 6(7): e1000097. doi:10.1371/journal.pmed1000097

Search strategy

Electronic search engine: Embase

Electronic databases searched: Embase and MEDLINE

Date of search: 08.10.2020

Search term: alzheimer AND ('intracerebral hemorrhage'/exp OR 'intracerebral hemorrhage' OR 'intracranial hemorrhage'/exp OR 'intracranial hemorrhage' OR 'brain hemorrhage'/exp OR 'brain hemorrhage' OR 'stroke'/exp OR stroke OR 'ischemic stroke'/exp OR 'ischemic stroke') AND ([article]/lim OR [article in press]/lim) AND ([english]/lim OR [german]/lim OR [italian]/lim OR [portuguese]/lim OR [spanish]/lim) AND [humans]/lim AND ([embase]/lim OR [medline]/lim) AND [1970-2020]/py AND [medline]/lim

Supplementary Table 1: Newcastle–Ottawa Quality Assessment Scale for Cohort Studies

Study name	Selection****	Comparability**	Outcome***	Overall score
Lee et al, 2020	****	**	***	9 / 9
Taipale et al, 2017	*-**	--	***	6 / 6
Vandenberghe et al, 2016	0-**	--	***	5 / 6
Benedictus et al, 2015	*-*0	--	***	5 / 6
Cummings et al, 2015	0-*0	--	00*	2 / 6
Salloway et al, 2014	0-**	--	0**	4 / 6
Chi et al, 2013	****	**	***	9 / 9
Tolppanen et al, 2013	****	**	***	9 / 9
Imfeld et al, 2013	****	**	***	9 / 9
Trzepacz et al, 2013	0-*0	--	00*	2 / 6
Epstein et al, 2012	0-*0	-	***	4 / 6
Lee et al, 2011	0-*0	--	00*	2 / 6
Li et al, 2010	*-**	--	***	6 / 6
Farlow et al, 2010	0-*0	--	00*	2 / 6
Grossberg et al, 2009	0-*0	--	00*	2 / 6
Richard et al, 2009	0-*0	--	0**	3 / 6
Clerici et al, 2009	0-*0	--	00*	2 / 6
Kessler et al, 2008	0-*0	--	0**	3 / 6
de Jong et al, 2008	0-*0	--	0**	3 / 6
AD2000 CG, 2008	0-**	--	0**	4 / 6
Bakchine et al, 2008	0-*0	--	00*	2 / 6
Mintzer et al, 2007	0-*0	--	00*	2 / 6
Howard et al, 2007	0-*0	--	00*	2 / 6
Soininen et al, 2007	0-*0	--	0**	3 / 6
Regan et al, 2006 (3962)	*-*0	--	0**	4 / 6
Silvestrini et al, 2006	0-*0	--	0**	3 / 6
Schneider et al, 2006	0-*0	--	0**	3 / 6
Seltzer et al, 2004	0-*0	--	00*	2 / 6
Imbimbo et al, 2000	0-**	--	00*	3 / 6
TOTAL	**51 / 91**	**8 / 8**	**56 / 87**	**115 / 186**

- : not applicable; 0: item not fulfilled

Supplementary Figure 1: Forest plot of the random effects meta-analysis for incident ischemic stroke (A), incident intracerebral hemorrhage (B) and incident stroke, all types (C) in patients with Alzheimer´s disease after exclusion of randomized controlled trials.

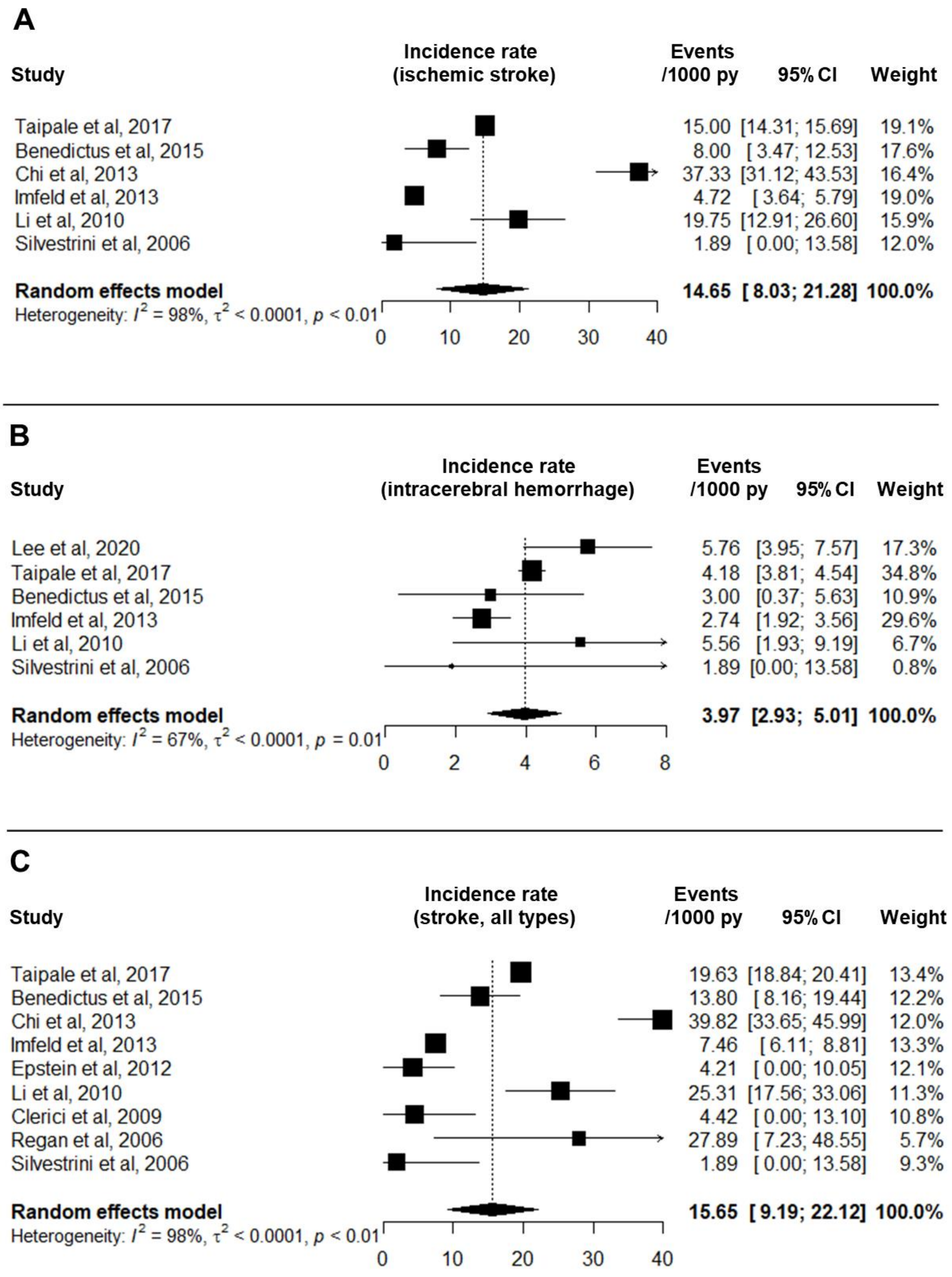

Supplementary Figure 2: Forest plot of the random effects meta-analysis for incidence rate ratios for ischemic stroke (A) and incident intracerebral hemorrhage (B) in patients with Alzheimer´s disease treated with antithrombotics compared with patients with Alzheimer´s disease not treated with antithrombotics.

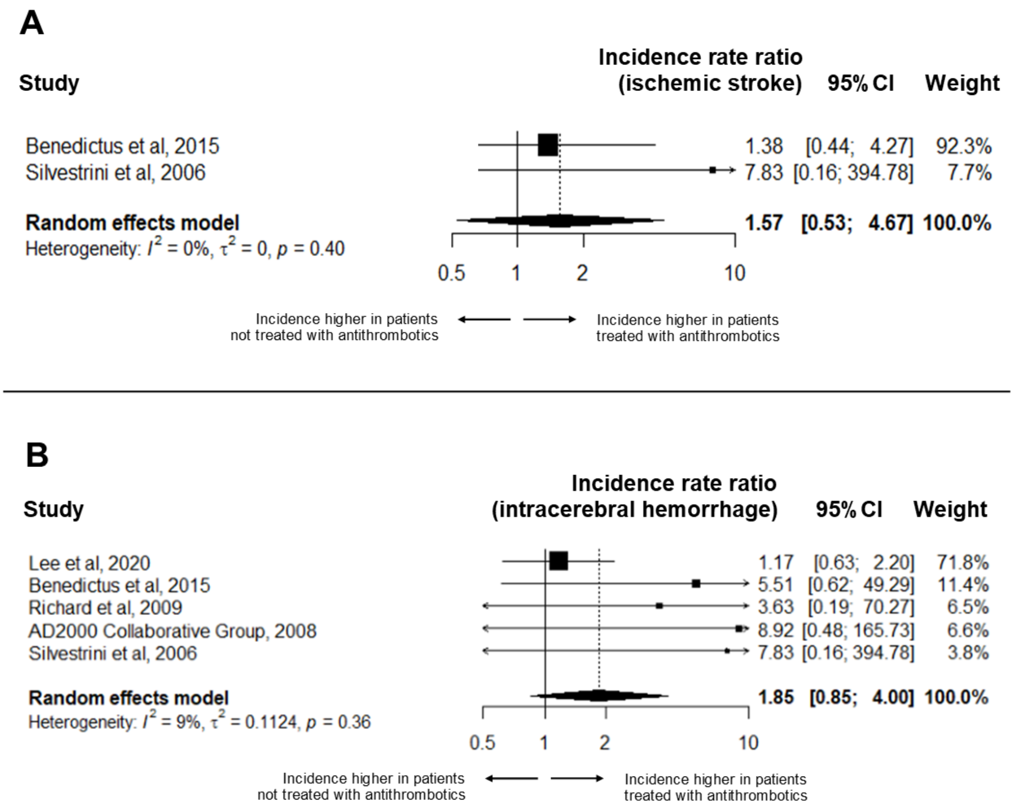

Supplementary Figure 3: Funnel plot for the meta-analysis of incidence rate ratios for ischemic stroke in patients with Alzheimer´s disease (compared to matched controls without AD)

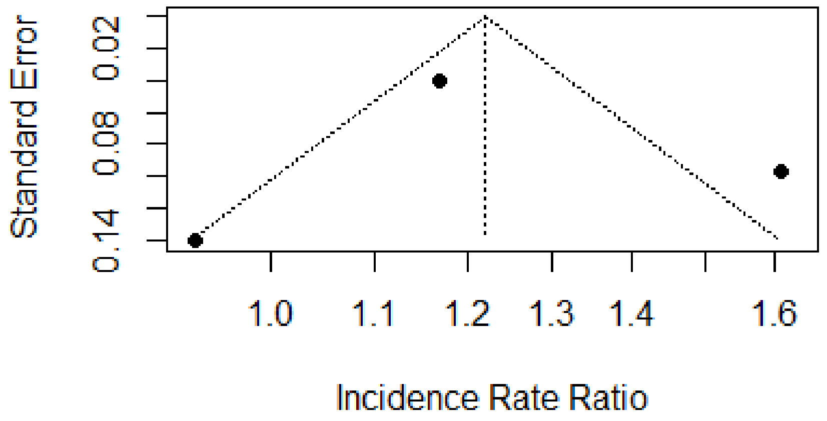

Supplementary Figure 4: Funnel plot for the meta-analysis of incidence rate ratios for intracerebral hemorrhage in patients with Alzheimer´s disease (compared to matched controls without AD)

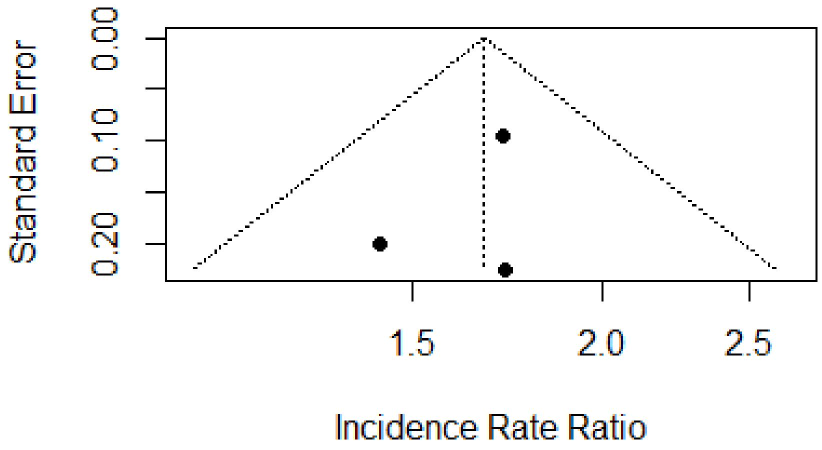

Supplementary Figure 5: Funnel plot for the meta-analysis of incidence rate ratios for stroke (all types) in patients with Alzheimer´s disease (compared to matched controls without AD)

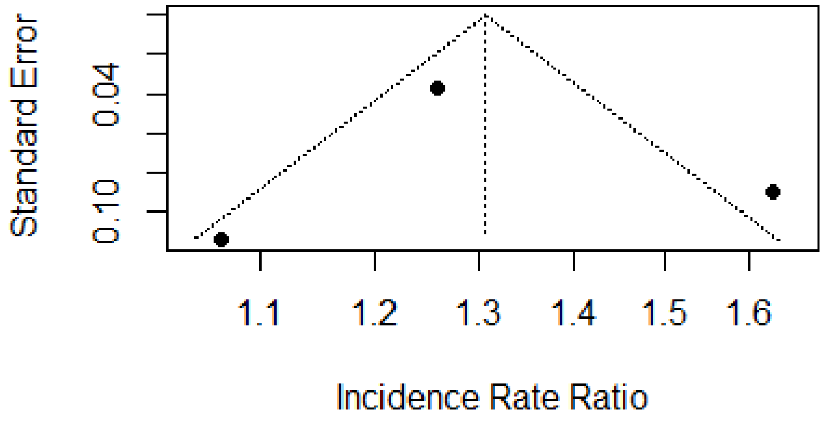

Supplement: Supplementary file 1 — Supplementary Information. [file 41598_2021_95821_MOESM1_ESM.pdf]
